# Diversity and composition of flower-visiting insects and related factors in three fruit tree species

**DOI:** 10.3897/BDJ.11.e100955

**Published:** 2023-09-08

**Authors:** Shoko Nakamura, Hisatomo Taki, Tomonori Arai, Ken Funayama, Shunsuke Furihata, Yuki Furui, Takamasa Ikeda, Hiromitsu Inoue, Kiyohiko Kagawa, Hidenari Kishimoto, Mitsuko Kohyama, Michiyo Komatsu, Akihiro Konuma, Ken Nakada, Suguru Nakamura, Nobuo Sawamura, Shoji Sonoda, Masahiro Sueyoshi, Seishi Toda, Katsuhiko Yaginuma, Shunsuke Yamamoto, Koki Yoshida, Tomoyuki Yokoi, Masatoshi Toyama

**Affiliations:** 1 Forestry and Forest Products Research Institute, Forest Research and Management Organization, Hachioji, Japan Forestry and Forest Products Research Institute, Forest Research and Management Organization Hachioji Japan; 2 Forestry and Forest Products Research Institute, Forest Research and Management Organization, Tsukuba, Japan Forestry and Forest Products Research Institute, Forest Research and Management Organization Tsukuba Japan; 3 Institute for Plant Protection, National Agriculture and Food Research Organization, Tsukuba, Japan Institute for Plant Protection, National Agriculture and Food Research Organization Tsukuba Japan; 4 Akita Fruit Tree Experiment Station, Yokote, Japan Akita Fruit Tree Experiment Station Yokote Japan; 5 Tottori Prefecture Horticultural Research Center, Hokueicho, Japan Tottori Prefecture Horticultural Research Center Hokueicho Japan; 6 Tohaku Agricultural Extension Center, Tottori Prefecture, Kotoura-cho, Japan Tohaku Agricultural Extension Center, Tottori Prefecture Kotoura-cho Japan; 7 Institute for Plant Protection, National Agriculture and Food Research Organization, Higashihiroshima, Japan Institute for Plant Protection, National Agriculture and Food Research Organization Higashihiroshima Japan; 8 School of Agriculture, Utsunomiya University, Utsunomiya, Japan School of Agriculture, Utsunomiya University Utsunomiya Japan; 9 Institute for Plant Protection, National Agriculture and Food Research Organization, Morioka, Japan Institute for Plant Protection, National Agriculture and Food Research Organization Morioka Japan; 10 Fruit Tree Research Institute, Uki, Japan Fruit Tree Research Institute Uki Japan; 11 Semboku Regional Development Bureau, Akita Prefecture, Daisen, Japan Semboku Regional Development Bureau, Akita Prefecture Daisen Japan; 12 Department of Business Development, National Agricultural Research Organization, Tsukuba, Japan Department of Business Development, National Agricultural Research Organization Tsukuba Japan; 13 Department of Agriculture, Forestry and Fisheries, Tottori Prefecture, Higashimachi, Japan Department of Agriculture, Forestry and Fisheries, Tottori Prefecture Higashimachi Japan; 14 Fukushima Agricultural Technology Centre, Fruit Tree Research Centre, Fukushima, Japan Fukushima Agricultural Technology Centre, Fruit Tree Research Centre Fukushima Japan; 15 Shimane Agricultural Technology Center, Izumo, Japan Shimane Agricultural Technology Center Izumo Japan; 16 Tea Research Insutitute, Kumamoto Prefecture, Mifune, Japan Tea Research Insutitute, Kumamoto Prefecture Mifune Japan; 17 Institute of Fruit Tree and Tea Science, National Agriculture and Food Research Organization, Morioka, Japan Institute of Fruit Tree and Tea Science, National Agriculture and Food Research Organization Morioka Japan; 18 Eastern Shimane Agriculture, Forestry and Fisheries Promotion Center, Izumo, Japan Eastern Shimane Agriculture, Forestry and Fisheries Promotion Center Izumo Japan; 19 Fukushima Agricultural Technology Centre, Koriyama, Japan Fukushima Agricultural Technology Centre Koriyama Japan; 20 Faculty of Life and Environmental Sciences, University of Tsukuba, Tsukuba, Japan Faculty of Life and Environmental Sciences, University of Tsukuba Tsukuba Japan

**Keywords:** insect pollination, agriculture, *Apis mellifera*, meteorological conditions, bees, Diptera, Coleoptera

## Abstract

Animal-mediated pollination is an essential ecosystem service for the production of many fruit trees. To reveal the community composition of flower-visiting wild insects which potentially contribute to fruit production and to examine the effects of geographic location, local meteorological conditions and locally introduced domesticated pollinators on them, we investigated the community composition of insects visiting the flowers (hereafter, “visitors”) of apple, Japanese pear and Oriental persimmon for 1‒3 years at 20 sites around Japan. While most of the variation (82%) of the community composition was explained by tree species with a slight contribution by geographic distance (2%), maximum temperature and tree species contributed 62% and 41% of the variation in total abundance of the visitors, respectively. Though the dominant families of the visitors varied spatiotemporally, the community composition of the visitors of apple and Japanese pear clearly differed from that of Oriental persimmon. While Andrenidae and Syrphidae together accounted for 46%‒64% of the visitors of apple and Japanese pear, Apidae represented 57% of the visitors of Oriental persimmon. The taxonomic richness, diversity and evenness of the visitors were best predicted by locally introduced domesticated pollinators and local meteorological conditions of wind speed and maximum temperature. Amongst these selected factors, locally introduced domesticated pollinators could have the largest impact. It seemed to be strongly related to the reduction of taxonomic richness, diversity and evenness of the visitors, accounting for 41‒89% of the variation. Results suggested that the community composition and total abundance of potential pollinators were predominantly determined by tree species and temperature, but locally introduced domesticated pollinators could have a determinantal pressure on the taxonomic diversity of the community.

## Introduction

Pollination is an important ecosystem service provided by flower-visiting animals (Gallai et al. 2009, Ollerton et al. 2011, IPBES 2016). Approximately 70% of the major crop species are supported by animal pollination with varying dependency (Klein et al. 2007). Amongst these crops, fruit trees are highly dependent on pollinating animals. About 42% of the world’s leading fruits “essentially” or “greatly” depend on animal pollination owing to their self-incompatible, dioecious or monoecious breeding systems. Meanwhile, only 22% of vegetables are pollinator-dependent (Klein et al. 2007). The economic value of animal pollination for fruit trees has been estimated to be approximately €51 billion, contributing to 23% of global fruit production (Gallai et al. 2009). It represents one-third of the global economic value of animal pollination (€153 billion). With recent worldwide declines of wild insects as the primary pollinators for crops (Potts et al. 2010, Goulson et al. 2015, IPBES 2016, Herrera 2019), fruit production may be especially at risk. Gallai et al. (2009) predicted that the production of fruits, along with vegetables and stimulant crops would be below the current consumption after pollinator loss. Meanwhile, production areas for fruit trees including shrubs are increasing. This is due to the increasing globalisation of the food trade, followed by diversification in the human diet and the larger incentives provided by the higher market values for fruit (Aizen et al. 2008, Lautenbach et al. 2012, Aizen et al. 2019). Conservation of wild insect pollinators is urgently needed to ensure sustainable fruit production.

Recent studies have emphasised the importance of complementary pollination by diverse insect pollinators (Garibaldi et al. 2013, Rader et al. 2016, Bernauer et al. 2022). Functionally diverse pollinators could increase production of crops such as pumpkin, apple, sweet cherry, almond and oilseed rape (Hoehn et al. 2008, Alomar et al. 2018, Eeraerts et al. 2019, Woodcock et al. 2019, Weekers et al. 2022b). However, the composition and diversity of the community of flower-visiting insects, i.e. potential pollinators, would vary amongst crop species, reflecting species-specific choice for flowers (Moeller 2005). The geographic and annual turnover of species and the interaction between introduced pollinators create complex spatiotemporal variation amongst the communities of flower-visiting insects even on the same plant species (Klein et al. 2003, Winfree et al. 2018). The negative effects of introduced pollinators on flower-visiting wild insects have recently been emphasised (Lindström et al. 2016, Weekers et al. 2022a). Faced with accelerating climate change, conserving the diverse pollinator community is increasingly important because it is linked to the stability and robustness of pollination services (Kühsel and Blüthgen 2015, de Bello et al. 2021). Since climate change is accompanied by increased climatic variability at local and short temporal scales, understanding the responses of communities of these insects and each taxon to local meteorological factors is highly important for entomophilous crop production (Burkle and Alarcon 2011, González-Varo et al. 2013, Moss and Evans 2022). However, the extent to which each of the factors contributes to the variation in the composition, diversity and abundance of the communities of flower-visiting insects is still not fully understood (Burkle and Alarcon 2011).

The introduction of domesticated pollinators, *Apis mellifera *or *Osmia cornifrons* began in apple orchards in Japan in the 1950s, following decreased fruiting success likely from the shortage of wild insect pollinators (Kobayashi 1979). However, information on the community composition of these wild pollinators contributing to Japanese crop production has been limited and, thus, the composition and abundance of flower-visiting insects were never examined in a comprehensive manner around Japan (Kobayashi 1979, Konuma and Okubo 2015, Kamo et al. 2022). Wild insect pollinators contribute to three-quarters of Japanese agricultural production and the production of pollinator-dependent crops has been gradually increasing (Konuma and Okubo 2015, Yoshiyama and Kimura 2018). The Japanese Ministry of Agriculture, Forestry and Fisheries has led this increase in the production of fresh fruits including entomophilous apples, Japanese pears, Oriental persimmons, peaches and plums, with the aim of increasing productivity by approximately 10% over the next 12 years (Japanese Ministry of Agriculture Forestry and Fisheries 2020). Considering fruits from trees comprise approximately 59% of production undertaken through pollinator-dependent open-field culture in Japan (Konuma and Okubo 2015), understanding potential wild pollinators or flower-visiting insects of each fruit tree species, their spatiotemporal variation and their responses to local meteorological factors are an urgent issue for developing a reasonable measure to conserve local flower-visiting insects in the context of sustainable agriculture in Japan.

We aimed to answer the following questions to understand potential pollinators of major entomophilous fruit trees in Japan, namely, apple, Japanese pear and Oriental persimmon: 1) How does the community composition of flower-visiting insects (hereafter, “visitors”) differ amongst the fruit tree species? 2) How do local meteorological conditions and the introduction of domesticated pollinators influence the composition and abundance of the communities of visitors and how large are these effects? 3) How does each taxonomic group of visitors respond to these factors?

## Materials and Methods

### Plant species

We selected apple (*Malus pumila* Mill.), Japanese pear (*Pyrus pyrifolia* (Burm.f.) Nakai) and Oriental persimmon (*Diospyros kaki *Thunb.) as targets for monitoring visitors. The apple and Japanese pear produce hermaphrodite, but self-incompatible flowers and the Oriental persimmon produces monoecious entomophilous flowers. The fruits are relatively familiar to Japanese people, with the top three production volumes of 763,300 (33% of the total production of fruit trees in Japan), 170,500 (7%) and 193,200 (8%) tonnes for apple, Japanese pear and Oriental persimmon, respectively, amongst the entomophilous fruits (Ministry of Agriculture Forestry and Fisheries 2020).

### Study sites

**Figure 1.**
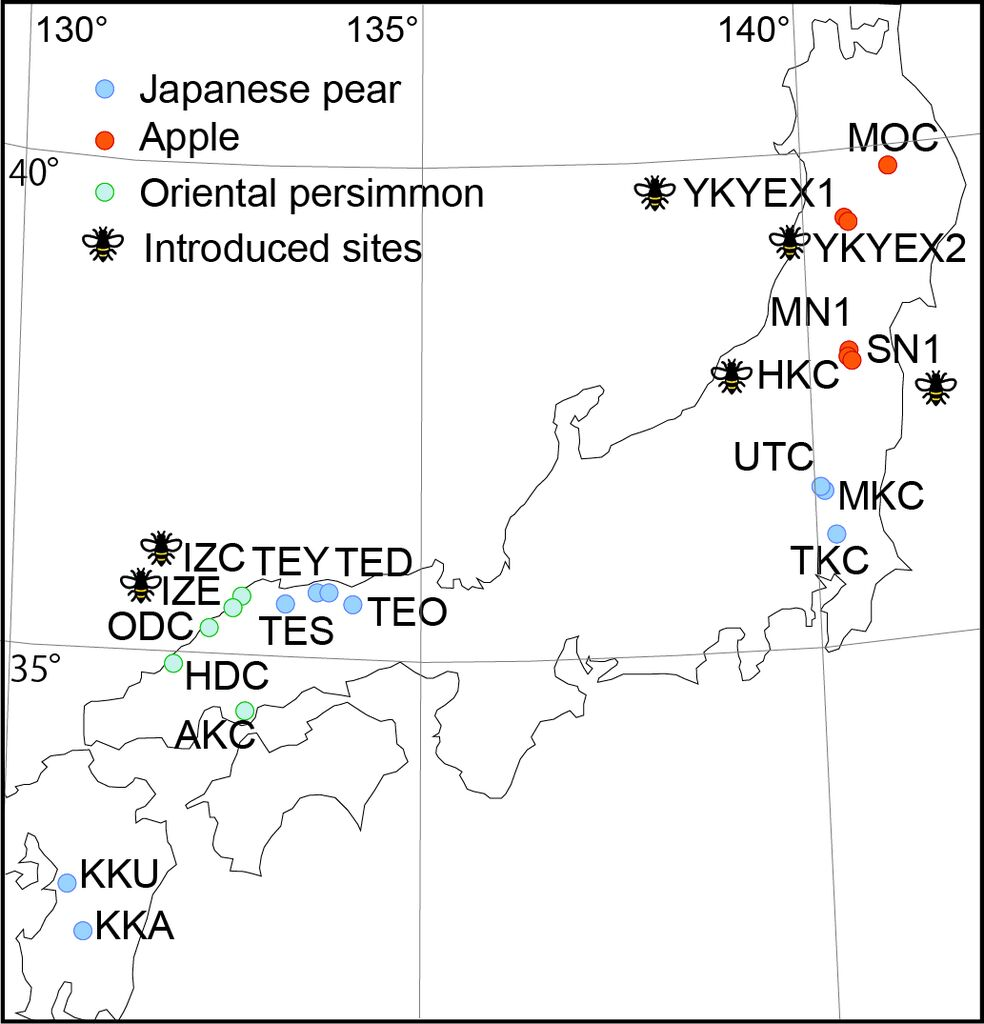
Locations of sites in Japan. The site names conform to Table S1: Suppl. material 1.

The surveys were conducted in orchards at 20 sites distributed in nine prefectures in Japan. The sites were within the Japanese agricultural landscape, with apples being grown at six sites (MOC, YKYEX1, YKYEX2, MN1, SN1, HKC), Japanese pear being grown at nine sites (UTC, MKC, TKC, TEO, TED, TEY, TES, KKU, KKA) and Oriental persimmon being grown at five sites (IZC, IZE, ODC, HDC, AKC) (Table S1: Suppl. material 1, Fig. 1). Most Japanese farms, including the orchards in this survey, are relatively small (< 2 ha), with 40% located in a Satoyama landscape comprising small mosaics of secondary woodlands, irrigation ponds, rice paddies, pastures and grasslands (Secretariat of the Convention on Biological Diversity 2010, McKinsey & Company 2016). The orchards studied were managed following local pest control practices, except there were no insecticides applied in one of the fields (MOC). Insecticide applications were limited to the non-blooming season at all the other sites. Artificial nests of domesticated pollinators, *Apis mellifera* and/or *Osmia cornifrons, *were introduced at six sites (hereafter, “introduced sites”). The distances between sites ranged from 2.6–1,247 km. The 30-year averages for the ten-day average daily air temperature during antheses were 10.8℃‒15.3℃ (minimum average: 4.8℃‒11.3℃; maximum average: 16.4℃‒20.4℃) at the sites of Japanese pear, 11.3℃‒15.9℃ (min: 4.9℃‒10.6℃; max: 17.8℃‒22.0℃) at the sites of apple and 18.5℃‒19.1℃ (min: 12.6℃‒14.7℃; max: 23.3℃‒24.8℃) at the sites of Oriental persimmon (Table S2: Suppl. material 2). The total precipitation ranged from 27.0–54.3 mm/10 days (Japan Meteorological Agency 2020). Flowering took place in late May to early April for the Japanese pear, a month later for the apple and a further two weeks later for the Oriental persimmon.

### Monitoring flower visitors and identification

Monitoring surveys were conducted on the days of full bloom (late March to late May) without rain at each site for one to three years, 2017–2019, covering morning to afternoon (Table S1: Suppl. material 1). To examine the community composition of the visitors, 1‒7 researcher(s) captured winged insects visiting the flowers of the fruit trees using plastic vials (50 cc in volume) by gently placing them over the visitors while we walked around the sites. We confirmed access by these visitors to the reproductive organs of the flowers or that they entered the flowers from the front, representing a high likelihood of successful pollination. We treated visitor assemblages per site per year as a “community”. The total minutes of sampling effort ranged from 30–5,400 min in 1–6 days per site per year. Ants and the domesticated pollinators, namely *A. mellifera* and *O. cornifrons,* were excluded from the surveys. However, the domesticated pollinators were captured during surveys in 2017 to check the proportions present at the sites.

We preserved the visitors captured during the surveys in 99.9% ethanol or 98% propylene glycol. The major flower visitors, namely Hymenoptera, Diptera and Coleoptera, were morphologically identified to family level and the others were identified to order level (hereafter, “taxon” for these identification units) using stereoscopic binoculars (Nikon SMZ800, Nikon Corporation, Tokyo, Japan). The top three taxa (bees: Andrenidae and Apidae; hoverflies: Syrphidae) and the most abundant coleopteran family, Scarabaeidae, were identified to the genus level. We did not identify visitors to the species level because functional diversity often explains ecosystem functioning including pollination better than species diversity (Gagic et al. 2015, Woodcock et al. 2019). Furthermore, the phylogenetic diversity could be translated to the diversity of ecosystem functions (Srivastava et al. 2012, Gagic et al. 2015). All the voucher specimens have been deposited at the National Institute of Agro-Environment Science, Tsukuba (NIAES).

### Meteorological data

We extracted daily-local meteorological data of maximum and minimum temperature, precipitation, sun duration and average wind speed from the nearest weather station (Table S3: Suppl. material 3, Table S4: Suppl. material 4). Seven sites had their own weather station, thus the distances to the weather station ranged from 0‒27.7 km (mean = 6.2 km, median = 2.8 km).

### Analysis

We conducted the following analyses to explore the factors related to the composition and abundance of the visitor communities and the abundance of each taxon. All statistical analyses were conducted in R version 4.1.0 (R Core Team 2021) using RStudio (RStudio Team 2021).

#### Community composition

The abundance records, based on family (Hymenoptera, Diptera and Coleoptera) or order (other taxa) identification of the visitors, were pooled for each site–year combination to construct the visitor communities. After excluding small communities (n < 50) and sites where the survey was conducted on only one day, we removed the taxa with relatively rare occurrences. These were taxa represented by only one individual across all the sites and years sampled. These reductions were to remove possible sampling bias and to compare communities, based on the taxa that potentially contribute to pollination. Rare taxa would have quite less contribution to pollination due to their low abundances. We then performed abundance-based rarefaction, rarefying each community to 51 individuals and used the data for the subsequent community analysis. According to the rarefaction analysis, these communities encompassed 83%‒99% of the taxa richness expected in these communities (Fig. S1: Suppl. material 8).

#### Factors related to community composition

We calculated the Bray–Curtis distances, based on the log-transformed community data and performed non-metric multi-dimensional scaling (nMDS) to visualise the differences amongst the communities. Log-transformation allows us to reduce the overappreciated large effect by dominant taxa (Borcard et al. 2018). To examine the effects of species of fruit trees, local meteorological conditions (maximum temperature and average wind speed), introduction of domesticated pollinators, geographic location and year on the composition of visitor communities, we fitted a generalised least squares (GLS) model with a set of Maximum Likelihood population effects covariance structure by site (Clarke et al. 2002, Pinheiro et al. 2022). GLS accounts for the non-independence of pairwise distances (Jha 2015, Plue et al. 2019), which is suitable for the analysis of dissimilarity amongst samples. In the full GLS model, we included between-community pairwise Bray–Curtis distances as a response variable. The effects of the difference in tree species were included as a categorical variable of tree species combination, "within-species", "between apple and Japanese pear", "between apple and Oriental persimmon" and "between Japanese pear and Oriental persimmon". The distances derived from differences in maximum temperature, average wind speed, the status of introduction of domesticated pollinators, geographic location and year were also included as explanatory variables. For the distances between the status of introduction of domesticated pollinators and between year, we used Gower’s dissimilarity (Gower 1971). The distances of local meteorological conditions were calculated as Euclidian distances. In terms of the local meteorological variables, maximum temperature and average wind speed were selected considering the availability of datasets with no missing data and correlations amongst variables (Fig. S2: Suppl. material 9). Before calculating the Euclidian distances for these two meteorological variables, the daily values were averaged over the corresponding dates of the surveys of the visitors and were standardised to zero mean and unit variance using the “decostand” function in the R package “vegan” (Oksanen et al. 2020). The geographical distances between the sites were calculated as metre distances using the “distm” function in the R package “geosphere” (Hijmans 2019). The meteorological and geographical distances were log-transformed before fitting them to the GLS model. Model selection was carried out based on Akaike’s Information Criterion (AIC) for the full model.

The contribution of each variable to the overall variation of the response variable explained by the best model, which was interpreted as the proportion reflecting the relative importance of the variable in the model, was calculated using deviance partitioning. The exclusive contributions from each explanatory variable in the best model were calculated as:

{(deviance of the model in which the explanatory variable of interest was removed from the best model) - (deviance of the best model)} / {(deviance of the null model) - (deviance of the best model)}.

The value is not affected by the collinearity amongst other variables in the model (Legendre 1993, Alzaga et al. 2009). The sum of the amounts of variation explained by each variable often differs from the total amount explained by the best model, which is predominantly due to the interactions between variables and overlaid effects (Barbosa et al. 2005).

Given that tree species was selected in the best model, Dunnett's multiple comparisons were carried out to test for the differences between tree species as a post hoc test. The distances between communities of different tree species were compared against those within the same tree species using the "glht" function in the R package "multcomp" (Hothorn et al. 2023). 

#### Factors related to taxonomic diversity in communities

We examined the effects of geographic and local meteorological factors, tree species, introduction of domesticated pollinators and annual fluctuations on the following diversity indices for each community: taxonomic richness, taxonomic diversity (Shannon diversity) and taxonomic evenness (Pielou’s evenness). We fitted generalised linear models (GLMs) for these response variables for each community and conducted model selection using AIC. The full model included the explanatory variables of mean maximum temperature, squared mean maximum temperature, average wind speed and squared average wind speed as local meteorological factors. Latitude was included as a geographical factor and tree species, the status of introduction of the domesticated pollinators and year were included as categorical variables. We did not include longitude as an explanatory variable in the full model to avoid strong multicollinearity with latitude (Fig. S2: Suppl. material 9). The numeric variables for local meteorological conditions and latitude were scaled to zero mean and unit variance. For the model of taxonomic richness, we assumed negative binomial error distribution. Gamma error distribution with log link was assumed for the taxonomic diversity and evenness. The communities used for these analyses were the same as those used for the community dissimilarity analysis. The contribution by each explanatory variable selected was calculated using a similar procedure used for the community composition.

#### Abundance of visitors

To explore the factors affecting the abundance of all visitors and each taxonomic group, we constructed generalised linear mixed effect models (GLMMs) and conducted model selection based on AIC. For these models, we assumed negative binomial error distribution and the response variables were the abundance of all the visitors (total abundance) or those of each taxonomic group recorded each day. To account for the varying sampling efforts amongst sites and days, we used the offset term of effort (min) in each day. The explanatory variables were daily maximum temperature, squared daily maximum temperature, daily average wind speed, squared daily average wind speed, latitude, tree species, year and the status of introduction of domesticated pollinators. Site was included as a random effect. For the analyses of each taxonomic group, the zero data were limited to the possible pairs of tree-insect combinations that had been detected at least once throughout the three-year survey. This was to examine the effects of local meteorological conditions and status of introduction of domesticated pollinators on the abundance of visitors, while eliminating the strong effect of tree species. The numeric explanatory variables were scaled to zero mean and unit variance. In the analyses for the abundance of each taxon, families that were sufficiently abundant to appear within the accumulated proportion of 80% in either of the three tree species (hereafter, “major” families) were analysed separately. The remaining less abundant 65 taxa (hereafter, “minor” families or taxa) were analysed in one model with the taxonomic group being an additional random variable. The contribution by each explanatory variable selected was calculated using a similar procedure used for the community composition.

### Data resources

The data underpinning the analysis reported in this paper are deposited at GBIF, the Global Biodiversity Information Facility, https://doi.org/10.15468/he2hm9 (Nakamura et al. 2023). 

## Results

### Community composition

In total, we identified 5,411 individuals of wild visitors from 37 communities ("community" as assemblages per site per year) across 20 sites (Table S5: Suppl. material 5). Most of the individuals recorded were assigned to Diptera (45%, 2,425 individuals from 33 families), Hymenoptera (43%, 2,345 from 16 families) and Coleoptera (11%, 607 from 17 families). The others were Hemiptera (24 individuals), Lepidoptera (8), Neuroptera (1) and Psocodea (1). Domesticated *A. mellifera* was captured at most of the sites (8 out of 9 sites) during 2017, while *O. cornifrons* was recorded at one site (YKY1) where artificial nests had been set in the field. These domesticated pollinators accounted for 51 ± 18% (mean ± SD; n = 3; 32%–64%) of the visitors in the introduced sites, while they were recorded for 11 ± 16% (n = 6; 0%–41%) in the unintroduced sites.

The dominant families of wild visitors differed amongst communities, creating spatiotemporal variation in the community composition (Table S5: Suppl. material 5). In Japanese pear, the most dominant families within the community of wild visitors were Syrphidae (six communities; dominance 35%‒60%), Andrenidae (three communities; 37%‒80%), Empididae (three communities; 28%‒75%), Anthomyiidae (two communities; 23%‒36%) or Tenthredinidae (one community; 26%). Amongst these dominant families, only Andrenidae, Syrphidae and Empididae were detected across almost all the communities (12‒14 out of 15 communities). Halictidae was consistently recorded from 12 communities of Japanese pear though small in numbers. Within the communities of apple, the most dominant families were Andrenidae (five communities; 18%‒72%), Syrphidae (three communities; 50%‒79%), Empididae (two communities; 22%‒35%), Apidae (two communities; 18%‒28%) or Sciaridae (one community; 64%). Amongst them, Andrenidae and Syrphidae were regularly recorded in all communities, except for one with only 11 individuals in total (SN1 in 2019). In Oriental persimmon, the most dominant families were Apidae (seven communities; 28%‒88%), Elateridae (three communities; 34%‒100%) or Scatopsidae (one community; 28%), but Apidae and Scarabaeidae were recorded throughout the communities, except for two (HDC in 2018 and 2019) with small sample sizes (< 24).

The most dominant genus within the major family also varied amongst sites (Table S6: Suppl. material 6). The Andrenidae genus *Andrena* was consistently recorded throughout the sites excluding those with quite small sample sizes (n < 5) for Rosaceae (apple and Japanese pear) trees, but the dominance varied from 5% to 58%. The most dominant genus in Syrphidae on Japanese pear differed amongst sites (*Eristalis* in four sites, *Melanostoma*, *Syrphus*, *Eupeodes* and *Episyrphus* each in one site), though *Melanostoma* was consistently recorded in apple with highly variable dominance (5%‒69%). The dominance of *Bombus* on Oriental persimmon varied from 7%‒57%, though the genus was the most dominant, representing 56% of the Apidae visitors to Oriental persimmon. More than 98% of the *Bombus* on Oriental persimmon were *Bombus ardens ardens*.* Bombus* was absent or relatively rare on Japanese pear and apple flowers. *Gametis* consistently dominated in Coleoptera on Oriental persimmon, but the dominance varied from 5%‒31%, except for one site where it did not occur.

The rarefied data for the community and diversity analyses consisted of 26 communities, including 10 for Japanese pear, nine for apple and seven for Oriental persimmon (Table S7: Suppl. material 7). While Hymenoptera and Diptera were dominant, comprising more than 90% of the individuals on Japanese pear and apple, Hymenoptera and Coleoptera were dominant on Oriental persimmon (Table 1). The major families (accumulated proportion > 80%) on Japanese pear were Andrenidae (24.3%), Syrphidae (21.4%), Empididae (18.6%), Anthomyiidae (10.2%), Tenthredinidae (3.5%) and Scarabaeidae (3.3%). Those for apple were Andrenidae (34.6%), Syrphidae (29.4%), Halictidae (6.3%), Apidae (5.9%) and Empididae (5%) and those for Oriental persimmon were Apidae (57.4%), Scarabaeidae (14.0%), Halictidae (7.3%) and Elateridae (5.9%).

#### Factors related to community composition

**Figure 2.**
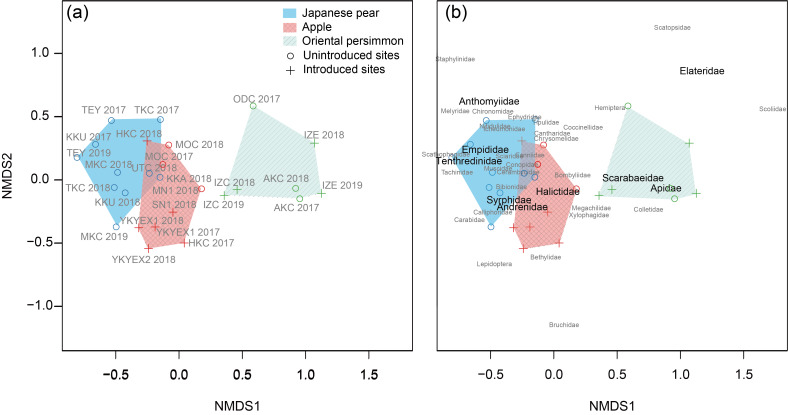
nMDS plot (a) with the names and years of the communities (information for each community is provided in Table S1: Suppl. material 1) and (b) with the names of related taxa. The taxon plot is based on the scores calculated as weighted averages of taxa for ordination configuration. The weighted average is constructed by weighing the abundance of each taxon to the corresponding value on the NMDS axes of each site. Taxa with black and larger fonts are major families. Blue: Japanese pear; red: apple; green: Oriental persimmon.

The difference in the community composition was best predicted by the geographic distance between sites and tree species (Table 2, Fig. 2). The community composition differed by tree species and the difference increased with the increasing difference in geographic distance. Deviance partitioning for the best model showed that tree species accounted for 82% of the variation explained by the best model, while geographic distance accounted for 2%. The post hoc test indicated that there was no significant difference in the community composition between apple and Japanese pear, while it clearly differed between either apple or Japanese pear and Oriental persimmon (Table 2). According to the taxon plot from nMDS, the communities of apple and Japanese pear were characterised by Anthomyiidae, Empididae, Tenthredinidae, Syrphidae, Andrenidae, Halictidae and most of the minor Dipteran families. The communities of Oriental persimmon were characterised by Scarabaeidae and Apidae (Fig. 2b).

### Taxonomic diversity and evenness

**Figure 3.**
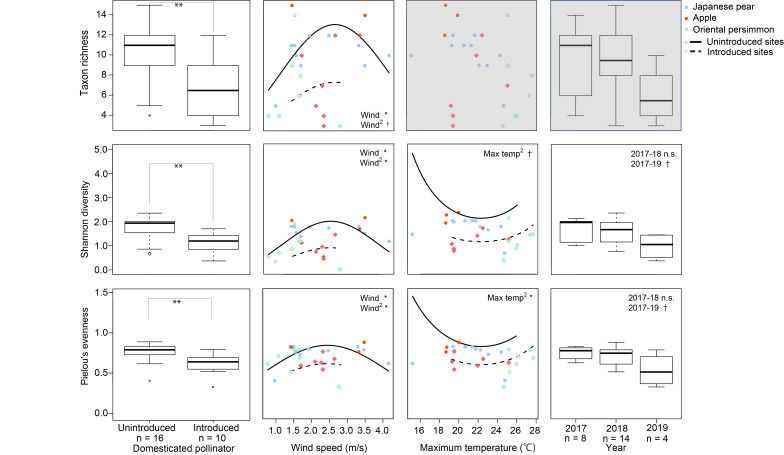
The responses of taxonomic richness, Shannon diversity and Pielou's evenness to the introduction of domesticated pollinators, average wind speed, maximum temperature and year. Panels of responses to the non-selected variables are shaded in grey. The predicted lines are drawn, based on the predictions for the year 2018. Richness, diversity and evenness calculations were based basically on family identification (see Materials and Methods for details). **: < 0.01, *: < 0.05, †: < 0.1

The average taxonomic richness in the communities, based mostly on the family identification, was 10.0 (5‒12 taxa) in Japanese pear, 9.1 (3‒15 taxa) in apple and 6.9 (3‒14 taxa) in Oriental persimmon. The average Shannon diversity in the communities was 1.7 (0.7‒2.0) in Japanese pear, 1.5 (0.8‒2.4) in apple and 1.2 (0.4‒2.1) in Oriental persimmon (Fig. 3). Pielou’s evenness in the communities was 0.7 (0.4‒0.8) in Japanese pear, 0.7 (0.6‒0.9) in apple and 0.6 (0.3‒0.8) in Oriental persimmon.

#### Factors related to taxonomic diversity in communities

The taxonomic richness in the communities was best predicted by introduction of domesticated pollinators and linear and quadratic terms of average wind speed (Table 3) and not by the factor of tree species. The taxonomic richness was lower at the introduced sites (6.7 ± 3.1 (mean ± SD) taxa, n = 10) than that of the unintroduced sites (10.2 ± 3.4 taxa, n = 16; Fig. 3). It was higher at sites with intermediate average wind speed than that of the other sites with lighter or stronger wind. Introduction of domesticated pollinators accounted for 88% of the variation explained by the best model, while the variables of average wind speed accounted for 44%.

Shannon diversity in the communities was best predicted by introduction of domesticated pollinators, linear and quadratic terms of average wind speed, quadratic term of maximum temperature and year (Table 3) and not by the factor of tree species. Shannon diversity was lower at the introduced sites than that at the unintroduced sites (Fig. 3). It was higher at sites with intermediate average wind speed, slightly higher at sites with higher mean maximum temperature than those of the other sites and tended to vary amongst years. Amongst the factors selected, introduction of domesticated pollinators accounted for 50% of the variation explained by the best model, with the variables of average wind speed accounting for 23%, the mean of the maximum temperature accounting for 18% and the year accounting for 22%.

Pielou's evenness in the communities was best predicted by similar explanatory variables to those in the best model for Shannon diversity (Table 3, Fig. 3) and not by the factor of tree species. Pielou’s evenness was lower at the introduced sites than those at the unintroduced sites. Pielou’s evenness was higher at sites with intermediate average wind speed and slightly higher at sites with higher mean maximum temperature than those of the other sites. It also varied amongst years. Introduction of domesticated pollinators accounted for 41% of the variation explained by the best model. Average wind speed accounted for 23%, mean maximum temperature accounted for 27% and year accounted for 32% of the variation.

### Abundance of visitors

#### Total abundance

Total abundance of visitors was best predicted by tree species and the quadratic term of maximum temperature (Table 4, Fig. S3: Suppl. material 10). A researcher captured 10.2 ± 9.0 (mean ± SD) visitors on Japanese pear (n = 37), 13.1 ± 14.4 visitors on apple (n = 46) and 4.8 ± 6.4 visitors on Oriental persimmon (n = 28) per 60 minutes of the survey. The maximum abundance was expected at around 22℃‒23℃, which was higher relative to the maximum daily temperature for normal years over the past 30 years (16.4℃‒22.0℃) at sites of apple and Japanese pear (Table S2: Suppl. material 2, Fig. S3: Suppl. material 10). However, the maximum temperature of maximum abundance was similar to or slightly lower than the maximum daily temperature for normal years at sites of Oriental persimmon (23.3℃‒24.8℃). The effect of maximum temperature accounted for 62% of the variation explained by the best model, while tree species accounted for 41%.

#### Abundance of each taxon

Based on the accumulated abundance (>80%), nine families, namely Andrenidae, Halictidae, Apidae, Tenthredinidae, Syrphidae, Empididae, Anthomyiidae, Scarabaeidae and Elateridae were determined to be major families and separately analysed. Each taxonomic group responded differently to the factors of tree species, local meteorological conditions and year (Table 4, Fig. S3: Suppl. material 10). Five out of nine major families responded to the factor of tree species. Andrenidae, Syrphidae and Halictidae were more abundant on the flowers of apple than on those of Oriental persimmon and were similarly abundant on the Rosaceae trees. Apidae abundantly visited flowers of apple and Oriental persimmon compared to Japanese pear. Elateridae was more abundant on Oriental persimmon than on apple and Japanese pear. The abundance of the minor taxa was not affected by tree species.

Either of the factors related to local meteorological conditions, namely maximum temperature and average wind speed were related to the abundance, except for Syrphidae and Empididae (Table 4). For taxa in which maximum temperature was selected in the best model, the abundance basically showed a unimodal response to temperature, except that the abundance of Elatreridae continued to increase and that of the minor taxa continuously decreased. Increasing average wind speed had no or continuous negative effect on the abundance of the visitors.

Introduction of domesticated pollinators had a negative or no clear effect, but never had a positive effect on the abundance of each taxon (Fig. S3: Suppl. material 10). Four major families, namely Halictidae, Anthomyiidae, Elateridae and Tenthredinidae and the minor taxa were negatively affected by the introduction of domesticated pollinators.

According to the deviance partitioning, the most contributing factor to the variation in the abundance of each taxon differed between taxonomic groups. Tree species contributed best to Andrenidae and Syrphidae; introduction of domesticated pollinators contributed best to Empididae, Anthomyiiade and Tenthredinidae; maximum temperature contributed best to Apidae, Halictidae and the minor taxa; average wind speed did best for Scarabaeidae; and year contributed best for Elatridae (Table 4).

## Discussion

We are the first to describe and compare the composition and abundance of visitor communities for three fruit tree species around Japan. The community composition, total abundance and taxonomic diversity of visitors varied amongst communities. This would have resulted from the varying responses of the taxonomic groups of the visitors to tree species, local meteorological conditions, local introduction of domesticated pollinators and/or other annually fluctuating factors.

### Community composition and total abundance

The community composition and total abundance were best explained by tree species and geographic distance between sites or daily maximum temperature. Though we must note that our sampling sites were geographically clustered by tree species, this result might imply that these factors could have considerable effects on the characteristics of the visitor communities, compared with the other variables examined including introduction of domesticated pollinators.

The visitor communities significantly differed between the Rosaceae species (apple and Japanese pear) and Oriental persimmon (Tables 1, 2). While Diptera dominated together with Hymenoptera on the Rosaceae species, they did not for Oriental persimmon. Abundance of Syrphidae (Diptera) was lower on the Oriental persimmon than on the other two tree species (Table 4). More than half of the visitors to Oriental persimmon were represented by Apidae with high dominance by *Bombus* species (Table 1). Kamo et al. (2022) also reported the dominance by a *Bombus* species amongst wild visitors to Oriental persimmon around Japan including an experimental orchard just next to our field site of Japanese pear at Ibaraki (TKC). It strongly supports our results that detected differences in the community composition amongst tree species. Such a consistent dominance by a single taxon was not detected in apple and Japanese pear. On Rosaceae flowers, the most dominant family spatiotemporally varied, ranging from Hymenoptera (Andrenidae, Apidae and Tenthredinidae) to Diptera (Syrphidae, Empididae, Anthomyiidae and Sciaridae) and their dominance also varied (18%‒80%, Table S5: Suppl. material 5). However, since many visits by hoverflies and bees to apple flowers have been reported (Pardo and Borges 2020), the contributions of these two groups are likely to be consistent worldwide.

This clear difference between the visitor communities of the two Rosaceae species and Oriental persimmon may have partially resulted from the responses of insects to different floral traits, such as shape and floral orientation (Rosaceae species: disc-shaped and multi-directional depending on the positions within inflorescences; Oriental persimmon: bell-shaped and downward), floral colour (Rosaceae species: white to pale pink; Oriental persimmon: greenish-pale yellow) or reward (Rosaceae species: hermaphrodite flowers provide both nectar and pollen; Oriental persimmon: monoecious flowers either of nectar or pollen). Phenological compatibility between the flowering and the active season of the visitors might also have played a key role (Rathcke and Lacey 1985, Campbell 2009, Schiestl and Johnson 2013, Morente-López et al. 2018). For example, the active season of the *Bombus* species was suitably matched with the flowering season of Oriental persimmon (Kamo et al. 2022), but was too early for the other two Rosaceae species, which bloom more than two weeks earlier. Spring emerging Andrenidae would be better suited to apple or Japanese pear than to Oriental persimmon (Motten 1986, Gardner and Asher 2006, Glaum et al. 2021). The substantial proportion of Diptera on apple and Japanese pear could be related to the early emergence of Syrphidae species (Bokina 2012, Moquet et al. 2015). Differences in the visitor composition between apple and pear have been reported, which are likely related to the different nutritional composition of the floral reward and scent of the flowers (Quinet et al. 2016, Smessaert et al. 2019). However, we did not detect any clear differences between them. This might be owing to our family-based analysis not being genus- or species-based, as our genus identification suggested the difference in the dominant genus in Syrphidae between apple and Japanese pear. However, our results still might suggest relative similarity in functional or ecological properties between the communities of apple and Japanese pear (Srivastava et al. 2012, Grab et al. 2019).

Geographic distance explained a relatively small proportion (2%) of the variation in the model for community composition, compared to tree species (82%; Table 2). Although previous studies reported community turnover along with geographic distance (Burkle and Alarcon 2011, Simanonok and Burkle 2014), our results suggested far more importance of tree species at this geographical scale (around Japan). However, daily maximum temperature explained more (62%) of the variation of the model of total abundance than explained by plant species (41%; Table 4). We could conclude that, although community composition was largely determined by tree species, local meteorological factors, i.e. local maximum temperature, could have a larger effect on total abundance of potential pollinators than tree species. Broken down to each taxon, eight of ten taxa responded to maximum temperature (Table 4, Fig. S3:  Suppl. material 10). Several studies have reported the responses of visitor abundance to temperature (Kühsel and Blüthgen 2015, Hamblin et al. 2018). Global warming or increasing events of extremely high temperature during antheses may affect the quality of pollination services, especially through the change in the number of visits by wild insects. However, the effect could differ between fruit trees. While a higher maximum temperature than normal may benefit the pollination of apple and Japanese pear by reaching the temperature of the highest visitor activity, it may decrease visitor activity in Oriental persimmon (Fig. S3:  Suppl. material 10).

### Taxonomic diversity in communities and abundance of each taxon

Taxonomic richness, diversity and evenness in the communities were not related to tree species, but to introduction of domesticated pollinators, average wind speed, maximum temperature and/or year (Table 3). Although the abundance of some families, namely Andrenidae, Syrphidae, Apidae, Halictidae and Elateridae, differed amongst tree species, the responses to tree species were not consistent amongst families, which might reflect varying sensory abilities and preferences (Campbell et al. 2010, Lázaro and Totland 2010, Montero‐Castaño and Vilà 2017, Wester and Lunau 2017, Ropars et al. 2022). These varying responses would have cancelled out the effect of tree species on the entire community. All of the three diversity indices were high at intermediate wind speeds (Fig. 3). Two of the major families (Apidae and Halictidae) were most abundant at the lowest average wind speed (Fig. S3: Suppl. material 10). Exclusion of other taxa by these dominant taxa at low wind speeds (though we did not examine interactions amongst wild visitors), decreased dominance of and reduced competitive exclusion by these dominant taxa at intermediate wind speeds and an additional reduction in the abundance of 65 minor taxa at higher wind speed (Fig. S3: Suppl. material 10) might have created this pattern, as predicted in the intermediate disturbance hypothesis (Hobbs and Huenneke 1992, Lazaro et al. 2016). Contrary to the responses to the wind speed, intermediate maximum temperatures seemed to have resulted in lower diversity and evenness. The higher dominance by at least four major families (Andrenidae, Apidae, Scarabaeidae and Halictidae), owing to the maximised visitation activities at intermediate temperature, might have resulted in decreased diversity and evenness of the communities at intermediate temperature.

Introduction of domesticated pollinators (mainly *A*. *mellifera*) was strongly related to the decrease in the taxonomic richness, diversity and evenness in the communities (41%‒88% of the variations explained; Table 3, Fig. 3). Smaller abundances of minor taxa at introduced sites would have caused this decrease (Table 4). Although we could not rule out the possibility that unexamined confounding factors like habitat quality affected visitations by these taxa (Kremen et al. 2007, Montero-Castaño and Vilà 2012), the potential of strong pressure from domesticated pollinators (Paini 2004, Thomson 2004, Fürst et al. 2014, Lindström et al. 2016, Weekers et al. 2022a) on the minor taxa rather than on the major taxa should be noted. Considering that the factor of introduction of domesticated pollinators explained quite large proportions of variations of diversity indices compared to the variables of wind speed, maximum temperature and year, the potential negative effect of introduced *A*. *mellifera* and other domesticated pollinators could be more pronounced than that of climate change, at least in a short period of time. Further studies comparing communities of flower visitors and their pollinating functions between pre- and post-introduction of domesticated pollinators would clarify the effects of domesticated pollinators on the quality and stability of pollination services by diverse wild insects (Garibaldi et al. 2013, de Bello et al. 2021).

### Implications for sustainable fruit production

Our analyses have shown that the composition of potential pollinator communities is largely determined by fruit tree species. This implies that preliminary conservation measures may be constructed, based on the knowledge of visitors to focal fruit tree species. However, given that our results have also suggested a spatiotemporal turnover of dominant taxa, conservation measures that are too specific, based on temporally and spatially limited observations, may possess a high risk of failure in the context of crop production. We have also highlighted the different levels of vulnerability to increasing temperature in terms of pollination services provided by wild visitors amongst crop species and potential harm from domesticated pollinators to the diversity of visitors which may be related to the stability of pollination services.

## Supplementary Material

3DD2AB69-629B-5B20-A58E-8C101FC0BA7B 10.3897/BDJ.11.e100955.suppl1 8331939 Supplementary material 1 Table S1 Locations, sampling methods, sampling efforts and status of introduction of domesticated pollinators at each site and year Data type Site data Brief description Locations, sampling methods, sampling efforts and status of introduction of domesticated pollinators at each site and year. Sampling efforts were converted to the minutes of the survey per person in the column "Effort". File: oo_897685.xlsx https://binary.pensoft.net/file/897685 Nakamura S, Taki H, Arai T, Funayama K, Furihata S, Furui Y, Ikeda T, Inoue H, Kagawa K, Kishimoto H, Kohyama M, Komatsu M, Konuma A, Nakada K, Nakamura S, Sawamura N, Sonoda S, Sueyoshi M, Toda S, Yaginuma K, Yamamoto S, Yoshida K, Yokoi T, Toyama M

FE9FDF14-592B-5AE2-9E9F-E1D167478BC2 10.3897/BDJ.11.e100955.suppl2 Supplementary material 2 Table S2 Normal weather conditions at the nearest weather station as the 30-year (1991–2020) average for every 10 days during antheses of Japanese pear, apple and Oriental persimmon from March to May Data type meteroorological data Brief description Normal weather conditions at the nearest weather station as the 30-year (1991–2020) average for every 10 days during antheses of Japanese pear, apple and Oriental persimmon from March to May. Figures with @ represent statistics that are not based on data for the 30 years, due to termination of meteorological observations. File: oo_870171.csv https://binary.pensoft.net/file/870171 Nakamura S, Taki H, Arai T, Funayama K, Furihata S, Furui Y, Ikeda T, Inoue H, Kagawa K, Kishimoto H, Kohyama M, Komatsu M, Konuma A, Nakada K, Nakamura S, Sawamura N, Sonoda S, Sueyoshi M, Toda S, Yaginuma K, Yamamoto S, Yoshida K, Yokoi T, Toyama M

F5E252E6-EFF0-566A-9417-C44252020BA9 10.3897/BDJ.11.e100955.suppl3 Supplementary material 3 Table S3 Locations of the nearest weather stations and the distances to the sites Data type location Brief description Locations of the nearest weather stations and the distances to the sites. File: oo_870174.csv https://binary.pensoft.net/file/870174 Nakamura S, Taki H, Arai T, Funayama K, Furihata S, Furui Y, Ikeda T, Inoue H, Kagawa K, Kishimoto H, Kohyama M, Komatsu M, Konuma A, Nakada K, Nakamura S, Sawamura N, Sonoda S, Sueyoshi M, Toda S, Yaginuma K, Yamamoto S, Yoshida K, Yokoi T, Toyama M

6AB960FC-0091-52FB-BFF5-CBB5364493D4 10.3897/BDJ.11.e100955.suppl4 Supplementary material 4 Table S4 Meteorological conditions of the survey days at each site Data type meteorological data Brief description Meteorological conditions of the survey days at each site. Data were extracted from the nearest weather station (see Table S3). File: oo_886571.csv https://binary.pensoft.net/file/886571 Nakamura S, Taki H, Arai T, Funayama K, Furihata S, Furui Y, Ikeda T, Inoue H, Kagawa K, Kishimoto H, Kohyama M, Komatsu M, Konuma A, Nakada K, Nakamura S, Sawamura N, Sonoda S, Sueyoshi M, Toda S, Yaginuma K, Yamamoto S, Yoshida K, Yokoi T, Toyama M

6A260969-B072-573E-9D4E-FD70A995D38A 10.3897/BDJ.11.e100955.suppl5 Supplementary material 5 Table S5 The numbers of visitors in each taxa collected from each site and year Data type occurrences Brief description The numbers of visitors in each taxa collected from each site and year. Light grey cells represent undetected taxa and orange-shaded cells represent the most dominant taxa within the community. Dark grey-shaded lines were communities with less than 50 individuals. *Apis mellifera* and *Osmia cornifrons* were only collected in the first year (2017). File: oo_870187.xlsx https://binary.pensoft.net/file/870187 Nakamura S, Taki H, Arai T, Funayama K, Furihata S, Furui Y, Ikeda T, Inoue H, Kagawa K, Kishimoto H, Kohyama M, Komatsu M, Konuma A, Nakada K, Nakamura S, Sawamura N, Sonoda S, Sueyoshi M, Toda S, Yaginuma K, Yamamoto S, Yoshida K, Yokoi T, Toyama M

965C2F23-B256-56A8-A254-C32DC5E7E5B4 10.3897/BDJ.11.e100955.suppl6 Supplementary material 6 Table S6 Genera detected in the families Andrenidae, Apidae, Syrphidae and Scarabaeidae and their proportions in the site-pooled communities Data type occurrences Brief description Genera detected in the families Andrenidae, Apidae, Syrphidae and Scarabaeidae and their proportions in the site-pooled communities. Grey cells represent undetected genera and orange, yellow, green or blue cells represent the most abundant genus in the families of Andrenidae, Apidae, Syrphidae or Scarabaeidae, respectively. File: oo_844678.xlsx https://binary.pensoft.net/file/844678 Nakamura S, Taki H, Arai T, Funayama K, Furihata S, Furui Y, Ikeda T, Inoue H, Kagawa K, Kishimoto H, Kohyama M, Komatsu M, Konuma A, Nakada K, Nakamura S, Sawamura N, Sonoda S, Sueyoshi M, Toda S, Yaginuma K, Yamamoto S, Yoshida K, Yokoi T, Toyama M

EA4C66F7-A01A-5F50-932F-12DE1519BB64 10.3897/BDJ.11.e100955.suppl7 Supplementary material 7 Table S7 Rarefied community composition, based on family or order identification Data type occurrences Brief description Rarefied community composition, based on family or order identification. This data were used for the community analysis. Grey cells represent undetected taxa and orange-shaded cells represent the most dominant taxa within the community. File: oo_870192.xlsx https://binary.pensoft.net/file/870192 Nakamura S, Taki H, Arai T, Funayama K, Furihata S, Furui Y, Ikeda T, Inoue H, Kagawa K, Kishimoto H, Kohyama M, Komatsu M, Konuma A, Nakada K, Nakamura S, Sawamura N, Sonoda S, Sueyoshi M, Toda S, Yaginuma K, Yamamoto S, Yoshida K, Yokoi T, Toyama M

889DB923-7F26-5EE7-B04B-4911CA7603C7 10.3897/BDJ.11.e100955.suppl8 Supplementary material 8 Figure S1 Rarefaction curves for the communities of visitors Data type images Brief description Rarefaction curves for the communities of visitors. The curves are drawn after excluding small communities (n < 50) and sites where the survey was conducted on only one day and removing the taxa with relatively rare occurrences. The vertical line represents 51 individuals, to which we rarefied these communities for the community analyses. The rarefied communities encompassed 83%‒99% of the taxa richness expected in these communities. File: oo_789414.pdf https://binary.pensoft.net/file/789414 Nakamura S, Taki H, Arai T, Funayama K, Furihata S, Furui Y, Ikeda T, Inoue H, Kagawa K, Kishimoto H, Kohyama M, Komatsu M, Konuma A, Nakada K, Nakamura S, Sawamura N, Sonoda S, Sueyoshi M, Toda S, Yaginuma K, Yamamoto S, Yoshida K, Yokoi T, Toyama M

EBD22D40-B97D-5233-B3F0-D77A3B8DE5E0 10.3897/BDJ.11.e100955.suppl9 8331943 Supplementary material 9 Figure S2 Correlation plots for the candidates of explanatory variables for meteorological factors Data type images Brief description Correlation plots for the candidates of explanatory variables for meteorological factors. File: oo_789413.pdf https://binary.pensoft.net/file/789413 Nakamura S, Taki H, Arai T, Funayama K, Furihata S, Furui Y, Ikeda T, Inoue H, Kagawa K, Kishimoto H, Kohyama M, Komatsu M, Konuma A, Nakada K, Nakamura S, Sawamura N, Sonoda S, Sueyoshi M, Toda S, Yaginuma K, Yamamoto S, Yoshida K, Yokoi T, Toyama M

15292B53-4A6F-53FE-B12F-8B620C42F090 10.3897/BDJ.11.e100955.suppl10 Supplementary material 10 Figure S3 The responses of visitor abundance to tree species, maximum temperature, wind speed, introduction of domesticated pollinators and year Data type images Brief description The responses of visitor abundance to tree species, maximum temperature, wind speed, introduction of domesticated pollinators and year. Figures in parentheses above the boxes of the boxplots represent sample sizes. Panels drawn for non-selected explanatory variables by the model selection are shaded in grey. The yellow shades in the panels for the responses to maximum temperature correspond to the maximum temperature range for normal years (16.4℃‒22.0℃ in apple and pear sites, 23.3℃‒24.8℃ in persimmon sites). File: oo_850899.pdf https://binary.pensoft.net/file/850899 Nakamura S, Taki H, Arai T, Funayama K, Furihata S, Furui Y, Ikeda T, Inoue H, Kagawa K, Kishimoto H, Kohyama M, Komatsu M, Konuma A, Nakada K, Nakamura S, Sawamura N, Sonoda S, Sueyoshi M, Toda S, Yaginuma K, Yamamoto S, Yoshida K, Yokoi T, Toyama M

## Figures and Tables

**Table 1.** Numbers and proportions of visitors pooled by tree species based on the rarefied communities. Families with asterisks represent major families, in which the accumulated proportion > 80% in each tree species.

**Order**	**Family**	**Japanese pear**				**Apple**				**Oriental persimmon**			
		n	%	Order Total n	Order Total %	n	%	Order Total n	Order Total %	n	%	Order Total n	Order Total %
Hymenoptera	Andrenidae	124	24.3 *	159	31.2	159	34.6 *	222	48.4	11	3.1	248	69.5
	Apidae	0	0.0			27	5.9 *			205	57.4 *		
	Halictidae	16	3.1			29	6.3 *			26	7.3 *		
	Tenthredinidae	18	3.5 *			0	0.0			0	0.0		
	Icheumonidae	0	0.0			4	0.9			1	0.3		
	Megachilidae	0	0.0			0	0.0			3	0.8		
	Bethylidae	1	0.2			1	0.2			0	0.0		
	Blasticotomidae	0	0.0			1	0.2			0	0.0		
	Colletidae	0	0.0			0	0.0			1	0.3		
	Figitidae	0	0.0			0	0.0			1	0.3		
	Scoliidae	0	0.0			1	0.2			0	0.0		
Diptera	Syrphidae	109	21.4 *	309	60.6	135	29.4 *	198	43.1	14	3.9	26	7.3
	Empididae	95	18.6 *			23	5.0 *			0	0.0		
	Anthomyiidae	52	10.2 *			3	0.7			1	0.3		
	Chironomidae	12	2.4			16	3.5			1	0.3		
	Ephydridae	12	2.4			1	0.2			2	0.6		
	Bibionidae	7	1.4			7	1.5			0	0.0		
	Calliphoridae	6	1.2			1	0.2			0	0.0		
	Muscidae	3	0.6			2	0.4			2	0.6		
	Sciaridae	4	0.8			1	0.2			1	0.3		
	Bombyliidae	0	0.0			4	0.9			0	0.0		
	Conopidae	1	0.2			2	0.4			0	0.0		
	Tachinidae	3	0.6			0	0.0			0	0.0		
	Tipulidae	1	0.2			0	0.0			2	0.6		
	Scatopsidae	0	0.0			0	0.0			2	0.6		
	Agromyzidae	1	0.2			0	0.0			0	0.0		
	Asilidae	0	0.0			1	0.2			0	0.0		
	Cylindrotomidae	0	0.0			1	0.2			0	0.0		
	Fanniidae	1	0.2			0	0.0			0	0.0		
	Sphaeroceridae	1	0.2			0	0.0			0	0.0		
	Tabanidae	0	0.0			0	0.0			1	0.3		
	Therevidae	1	0.2			0	0.0			0	0.0		
	Xylophagidae	0	0.0			1	0.2			0	0.0		
Coleoptera	Scarabaeidae	17	3.3 *	41	8.0	16	3.5	37	8.1	50	14.0 *	79	22.1
	Elateridae	1	0.2			0	0.0			21	5.9 *		
	Chrysomelidae	8	1.6			4	0.9			3	0.8		
	Cerambycidae	6	1.2			0	0.0			1	0.3		
	Coccinellidae	1	0.2			3	0.7			2	0.6		
	Melandryidae	0	0.0			6	1.3			0	0.0		
	Nitidulidae	3	0.6			3	0.7			0	0.0		
	Cantharidae	2	0.4			1	0.2			2	0.6		
	Melyridae	2	0.4			0	0.0			0	0.0		
	Pyrochroidae	0	0.0			2	0.4			0	0.0		
	Curculionidae	0	0.0			1	0.2			0	0.0		
	Oedemeridae	0	0.0			1	0.2			0	0.0		
	Staphylinidae	1	0.2			0	0.0			0	0.0		
Hemiptera		0	0.0	0	0.0	2	0.4	2	0.4	4	1.1	4	1.1
Lepidoptera		1	0.2	1	0.2	0	0.0	0	0.0	0	0.0	0	0.0
	Total	510	100.0			459	100.0			357	100.0		

**Table 2.** Results of model selection for the fitted generalised least squares (GLS) model for the distances between communities. The best model selected by AIC and the null model with AIC value are shown. The result of Dunnett's multiple comparison for testing differences in the community composition between different tree species and within the same tree species is also shown. Contributions of selected variables were calculated, based on the deviance partitioning. The full GLS model included Bray–Curtis distances, based on the log-transformed community data as a response variable, combination of tree species between the communities, distances separately derived from local meteorological conditions (maximum temperature and average wind speed), introduction of domesticated pollinators, geographic location and year were included as explanatory variables, with a set of Maximum-likelihood population effects covariance structure amongst sites (MLPE; (Clarke et al. 2002, Pinheiro et al. 2022)). For details about the methods for calculating each distance, please refer to the methods section.

		**Estimate**	**Std. Error**	**t-value**	**p-value**	**AIC**	**Contributions**
Null model						-286.23	
	Intercept	0.580	0.030	22.10	< 0.01		
Best model						-547.47	
	Intercept (Tree species combination = Within)	0.368	0.035	13.754	< 0.01		
	log (Geographic distance)	0.008	0.002	3.551	< 0.01		2%
	Tree species combination						82%
	Apple–Pear	0.028	0.014	1.949	0.052		
	Apple–Persimmon	0.187	0.018	10.277	< 0.01		
	Pear–Persimmon	0.309	0.016	18.894	< 0.01		
**Post-hoc (Dunnett's multiple comparison)**		** **	** **	**z-value**	**p-value**		
	Apple–Pear vs Within			1.949	0.135		
	Apple–Persimmon vs Within			10.277	< 0.01		
	Pear–Persimmon vs Within			18.894	< 0.01		

**Table 3.** The results of model selection for the generalised linear models (GLMs) for the response variables of taxonomic richness, Shannon diversity and Pielou's evenness. The best models selected by AIC, with the respective null models with AIC values are shown. The contributions of the variables selected were calculated, based on deviance partitioning. The full model included one of the three diversity indices derived from the rarefied community as a response variable and mean maximum temperature, squared mean maximum temperature, average wind speed, squared average wind speed, latitude, tree species, year and the status of introduction of domesticated pollinators as explanatory variables. We assumed negative binomial error distribution for the model of taxonomic richness and gamma error distribution with log-link was assumed for diversity and evenness.

		**Estimate**	**Std. Error**	**t-value**	**p-value**	**AIC**	**Contributions**
Richness	Null model					144.7	
	Intercept	2.180	0.081	26.80	< 0.01		
	Best model					138.6	
	Intercept (Domesticated bee = Unintroduced)	2.517	0.127	19.88	< 0.01		
	Domesticated bee	-0.578	0.167	-3.45	< 0.01		88%
	Wind	0.183	0.085	2.15	0.032		44%
	Wind^2	-0.153	0.079	-1.94	0.053		
Shannon diversity	Null model					48.3	
	Intercept	0.410	0.070	5.830	< 0.01		
	Best model					40.0	
	Intercept (Domesticated bee = Unintroduced, Year = 2017)	0.683	0.146	4.675	< 0.01		
	Domesticated bee	-0.613	0.179	-3.430	< 0.01		50%
	Wind	0.198	0.081	2.453	0.024		23%
	Wind^2	-0.214	0.100	-2.161	0.044		
	Temp^2	0.163	0.079	2.061	0.053		18%
	Year (2018)	0.070	0.142	0.495	0.626		22%
	Year (2019)	-0.444	0.225	-1.978	0.063		
Pielou's evenness	Null model					-22.0	
	Intercept	-0.348	0.038	-9.146	< 0.01		
	Best model					-32.7	
	Intercept (Domesticated bee = Unintroduced, Year = 2017)	-0.190	0.078	-2.449	0.024		
	Domesticated bee	-0.312	0.095	-3.272	< 0.01		41%
	Wind	0.092	0.043	2.149	0.045		23%
	Wind^2	-0.122	0.053	-2.307	0.032		
	Temp^2	0.111	0.042	2.627	0.017		27%
	Year (2018)	0.015	0.075	0.202	0.842		32%
	Year (2019)	-0.310	0.120	-2.587	0.018		

**Table 4.** The results for the generalised linear mixed effect models (GLMMs) for total abundance of visitors, abundances of major families and other minor taxa. The best models selected by AIC, with the respective AIC value of null models are shown. The figures are the estimates in the models and those in parentheses represent p-values. The full model included the response variable of total abundance of visitors or the abundance of each taxon for each day, with the offset term of sampling effort. The explanatory variables were the daily maximum temperature, squared daily maximum temperature, daily average wind speed, squared daily average wind speed, latitude, tree species, year and the status of introduction of domesticated pollinators and the random variable was site. We assumed a negative binomial error distribution for these models. For the model on the minor taxa, the random variable of taxonomic group was also included.

		**Intercept**	**Plant**	**Domestic bee**	**Max temp**	**Max temp^2**	**Wind**	**Wind^2**	**Year**	**AIC**	**n**
Total	Null	-2.211								1068.7	111
		(< 0.01)									
	Best	-1.584	Pear: -1.081			-0.241				1063.7	111
		(< 0.01)	(0.560)			(< 0.01)					
			Persimmon: -0.262								
			(0.026)								
Contributions			41%		62%						
Andrenidae	Null	-4.544								648.4	111
		(< 0.01)									
	Best	-2.944	Pear: -0.453		0.502	-0.333				623.7	111
		(< 0.01)	(0.463)		(< 0.01)	(0.024)					
			Persimmon: -4.262								
			(< 0.01)								
Contributions			62%		50%						
Syrphidae	Null	-4.573								632.7	111
		(< 0.01)									
	Best	-3.463	Pear: 0.043							599.8	111
		(< 0.01)	(0.850)								
			Persimmon: -3.39								
			(< 0.01)								
Contributions			100%								
Apidae	Null	-6.186								415.6	111
		(< 0.01)									
	Best	-4.755	Pear: -3.094		0.880	-0.812	-0.357			388.6	111
		(< 0.01)	(< 0.01)		(< 0.01)	(< 0.01)	(0.090)				
			Persimmon: 0.202								
			(0.849)								
Contributions			26%		66%		8%				
Empididae	Null	-6.23								341.3	83
		(< 0.01)									
	Best	-4.661		-16.862					2018: 0.812	316.9	83
		(< 0.01)		(0.933)					(< 0.01)		
									2019: -0.162		
									(0.806)		
Contributions				66%					34%		
Scarabaeidae	Null	-5.913								394.9	111
		(< 0.01)									
	Best	-5.214			0.673	-0.675	-0.804	-0.851		373.6	111
		(< 0.01)			(0.045)	(0.020)	(0.021)	(< 0.01)			
Contributions					37%		50%				
Halictidae	Null	-5.335								446.5	111
		(< 0.01)									
	Best	-3.814	Pear: -1.079	-1.338	0.568	-0.385	-0.311			429.9	111
		(< 0.01)	(0.118)	(0.038)	(<0.01)	(0.013)	(0.050)				
			Persimmon: -1.746								
			(< 0.01)								
Contributions			21%	12%	64%		14%				
Anthomyiidae	Null	-5.985								255.1	83
		(< 0.01)									
	Best	-3.987		-3.137		-0.500			2018: -1.265	246.1	83
		(< 0.01)		(< 0.01)		(0.134)			(0.060)		
									2019: 0.368		
									(0.680)		
Contributions				42%	15%				29%		
Elateridae	Null	-8.95								127.9	111
		(< 0.01)									
	Best	-8.765	Pear: -0.597	-2.435	0.371		0.559		2018: 0.560	119.5	111
		(< 0.01)	(0.679)	(0.036)	(0.031)		(0.144)		(0.397)		
			Persimmon: 3.148						2019: -2.057		
			(0.022)						(0.641)		
Contributions			40%	18%	15%		9%		43%		
Tenthredinidae	Null	-6.55								187.0	83
		(< 0.01)									
	Best	-5.588		-1.822		-0.478				183.2	83
		(< 0.01)		(0.039)		(0.084)					
Contributions				50%	47%						
Minor taxa	Null	-8.99								3171.7	4323
		(< 0.01)									
	Best	-8.077		-1.51	-0.188	-0.304		-0.158		3148.7	4323
		(< 0.01)		(0.040)	(0.090)	(< 0.01)		(< 0.01)			
Contributions				12%	57%		27%				
