# Supplementary figures and images for: Diversity and composition of flower-visiting insects and related factors in three fruit tree species
Source: Biodivers Data J. 2023 Sep 8;11:e100955. doi: 10.3897/BDJ.11.e100955 (PMC10504601; doi:10.3897/BDJ.11.e100955)

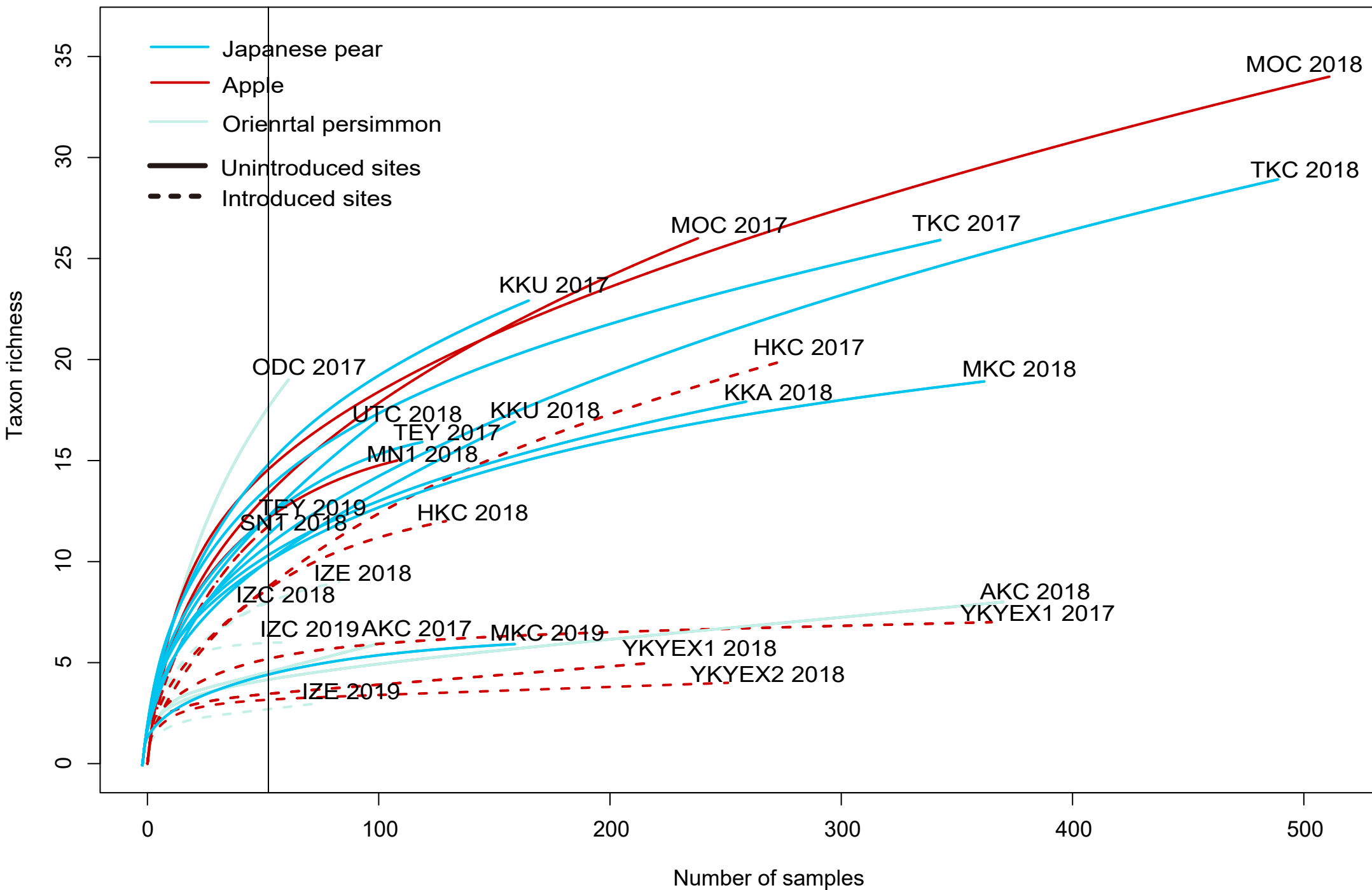

Supplement: Supplementary material 8 — Figure S1 Rarefaction curves for the communities of visitors [file bdj-11-e100955-s008.pdf]

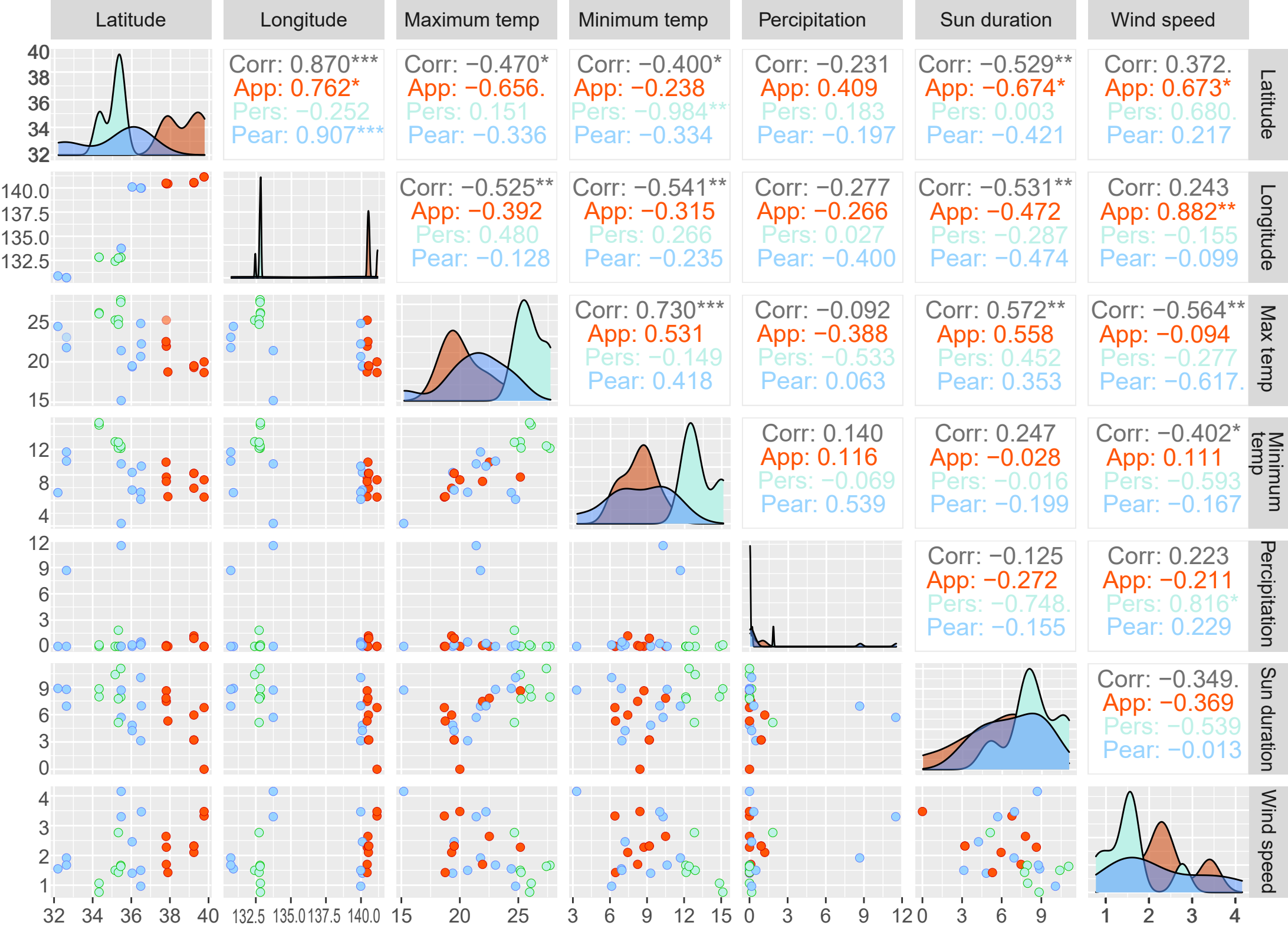

Supplement: Supplementary material 9 — Figure S2 Correlation plots for the candidates of explanatory variables for meteorological factors [file bdj-11-e100955-s009.pdf]

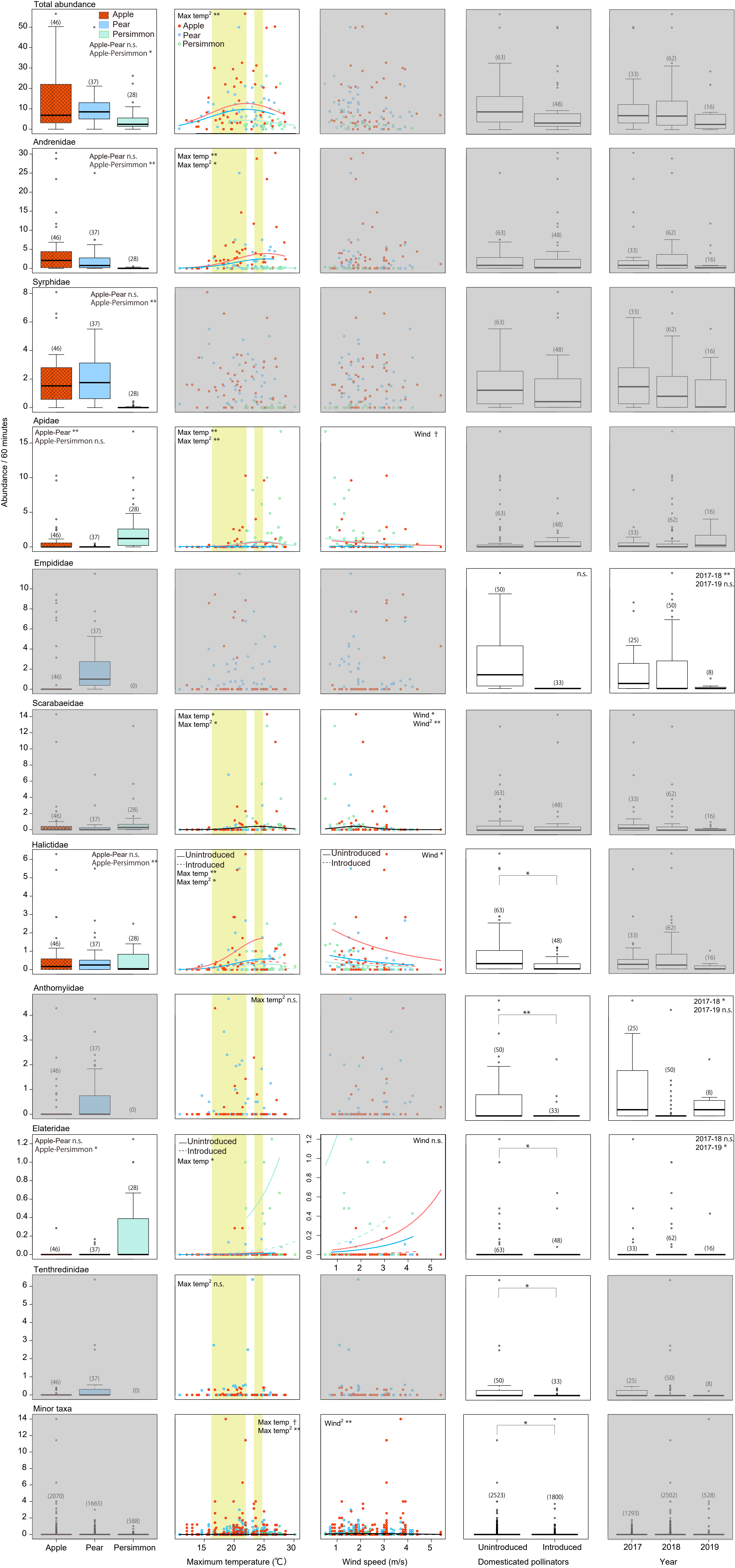

Supplement: Supplementary material 10 — Figure S3 The responses of visitor abundance to tree species, maximum temperature, wind speed, introduction of domesticated pollinators and year [file bdj-11-e100955-s010.pdf]
